# Supplementary material for: High Incidence of Intracerebral Hemorrhaging Associated with the Application of Low-Intensity Focused Ultrasound Following Acute Cerebrovascular Injury by Intracortical Injection
Source: Pharmaceutics. 2022 Oct 6;14(10):2120. doi: 10.3390/pharmaceutics14102120 (PMC9609794; doi:10.3390/pharmaceutics14102120)
Supplement: Supplementary file 1 [file pharmaceutics-14-02120-s001.zip › pharmaceutics-1920429-supplementary.pdf]

pharmaceutics

MDPI

Supplementary Materials: High Incidence of Intracerebral Hemorrhaging Associated with the Application of Low-Intensity Focused Ultrasound following Acute Cerebrovascular Injury by Intracortical Injection

Evgenii Kim, Jared Van Reet, Hyun-Chul Kim, Kavin Kowsari and Seung-Schik Yoo

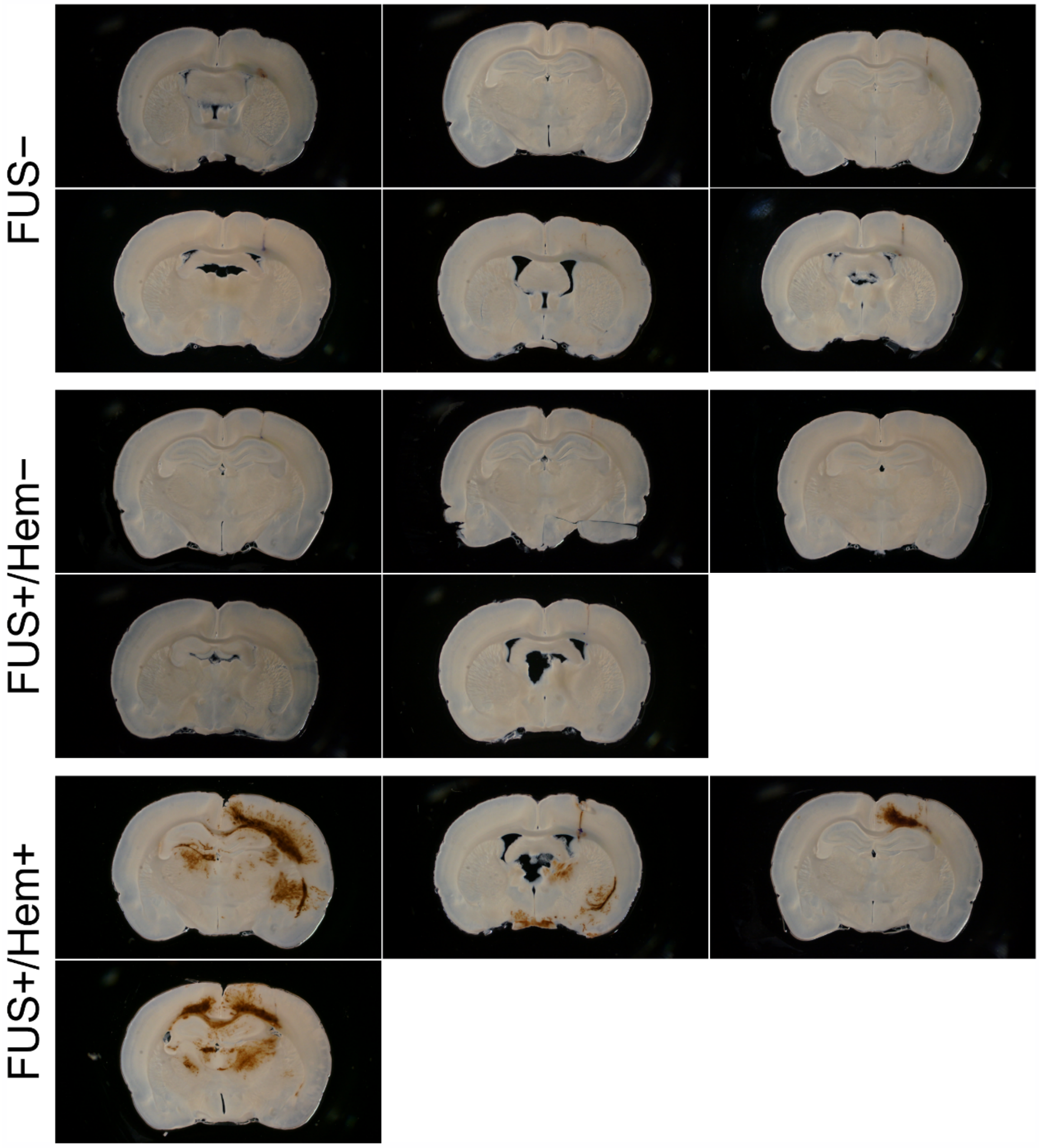

Figure S1. **Animal-specific Bright-field Images at the Injection Site.** The purple-brown hue visible at the injection site among FUS-/Hem- and FUS+/Hem- animals indicates the spatial distribution of OA and FITC-d in the needle path.
